# New T staging recommendations for recurrent nasopharyngeal carcinoma

**DOI:** 10.1007/s00432-024-05821-3

**Published:** 2024-06-08

**Authors:** Mingjing Zhu, Pian Li, Huisi Zhang, Lanhua Wu, Kang Min

**Affiliations:** 1 Department of Radiation Oncology, The First Affiliated Hospital of Guangxi Medical University, Nanning, Guangxi 530021 China; 2 Department of Oncology Radiotherapy, The First Affiliated Hospital, Hengyang Medical School, University of South China, Hengyang, Hunan Province 421001 China

**Keywords:** Recurrent nasopharyngeal carcinoma, T staging, Medial pterygoid muscle

## Abstract

**Objective:**

The International Union for Cancer Control/American Joint Committee on Cancer (UICC/AJCC) rT staging is not clinically practical for recurrent nasopharyngeal carcinoma (rNPC). The aim of this study was to establish a new rT staging to guide the treatment of rNPC.

**Methods:**

We conducted a retrospective analysis of 175 patients diagnosed with rNPC between January 2012 and December 2020, using ROC curve analysis to evaluate its effectiveness.

**Results:**

We analyzed the overall survival (OS) and progression-free survival(PFS) of patients diagnosed with rNPC according to the 8th (UICC/AJCC) rT staging, and found that the overall survival of rT1 and rT2 patients (OS; 29.98% vs. 27.09%, *p* = 0.8059) and progression-free survival (PFS; 28.48% vs. 26.12%, *p* = 0.4045) had no significant difference. In rT1 and rT2 patients of this study, overall survival(OS; 30.44% vs. 24.91%, *p* = 0.0229) and progression-free survival(PFS 29.12% vs. 24.03%, *p* = 0.0459) had a significant difference. Smoking, family history, and time interval of initial recurrence were independent prognostic factors for OS and PFS.

**Conclusion:**

The new rT staging of this study has a better predictive value for survival of rNPC patients than the 8th (UICC/AJCC) rT staging.

## Introduction

Nasopharyngeal carcinoma (NPC) is a highly invasive malignant tumor with an extremely uneven geographical distribution throughout the world (Tang et al. 2016). NPC is more common in southern China, especially Guangxi Province. According to Data released in 2020, the incidence and mortality of NPC in Guangxi Province is significantly higher than the national average (2.67/100,000 and 1.31/100,000), the incidence of nasopharyngeal cancer in Guangxi was 10.71/100,000, and the mortality was 5.15/100,000 (Li et al. 2020). The standard treatment for NPC is radiotherapy (RT), with or without chemotherapy, depending on the stage of the disease. Although the combination of radiation therapy and combined therapy can achieve good control, about 10-15% of patients have residual or recurrent primary and/or local sites after treatment (Mao et al. 2016, Zhang et al. 2015, Liu et al. 2022, Chen et al. 2022).

The treatment of rNPC can be challenging, and OS results after tumor recurrence are poor. Secondary radiotherapy is the main treatment for rNPC. Five-year survival rates have been reported to range from 28 to 60%, depending on the stage of disease (Zhou et al. 2021). Chemotherapy, molecular targeting and immunotherapy are also available options (Zong er al. 2021). In addition, surgical treatment, including neck lymph node dissection and nasopharyngeal lesion resection, is another treatment option ( Liu et al. 2019, Tang et al. 2019). The above treatment procedures should be carried out under the guidance of an accurate staging system.

NPC is classified according to the UICC/AJCC tumor lymph node metastasis (TNM) staging system. The system, which was updated in 2016 to its 8th edition (6th edition 2002, 7th edition 2009), classifies primary diseases and lymph node diseases. This staging system is commonly used to predict the prognosis of such patients after radiotherapy, to guide clinicians in making treatment decisions for different risk groups, and to evaluate treatment outcomes between different centers. However, these rT staging systems have great clinical impracticality, as most studies have found little difference in survival among patients with different T stages (Liang et al. 2016, Kang et al. 2017, Li et al. 2023, Kang et al. 2017). Therefore, there is an urgent need for a new rT staging system to guide the treatment of rNPC.

## Theme and method

### Patients selection

We retrospectively analyzed 175 patients with rNPC treated with IMRT at The First Affiliated Hospital of Guangxi Medical University between January 2012 and December 2020. All patients underwent detailed clinical and laboratory examinations, head and neck magnetic resonance imaging (MRI), nasopharyngoscopy biopsy, chest X-ray or CT, abdominal ultrasound or CT, and bone scans to confirm the presence of distant metastases. Inclusion criteria: (1) no secondary primary tumor; (2) No distant metastasis before treatment; (3) Recurrent nasopharyngeal carcinoma was confirmed by pathology or imaging; (4) Complete image information can be obtained. The study was approved by the ethics Review Committee of Guangxi Medical University.

### Clinical staging

All MRI images were independently reviewed by two radiologists who specialize in head and neck MRI. Any disagreements are resolved by consensus, with reference to the relevant patient clinical information (e.g. cranial nerve palsy, lymph node size, etc.). Tumors were staged according to the 8th UICC/AJCC staging system. In addition, this study focused on the re-staging of the medial pterygoid muscle(MP). After completion of treatment, patients were evaluated every 3 months for the first two years, every 6 months for the subsequent one years, If there is no follow-up on time, we would follow up with clinic visits, phone interviews or written letters. The information obtained was used to assess patient survival, recurrence patterns, and incidence of distant metastases. Follow-up tests include chest x-rays or CT scans, liver and abdominal ultrasonography, whole-body bone scans, head and neck CT or MRI, and fibroscopy with or without biopsy.

### Statistical analysis

Follow-up time was defined as the date of completion of treatment at our facility up to the date of death or last contact. OS is the time from initiation of radiotherapy to all-cause death. PFS is defined as the time from treatment to tumor progression (local, regional recurrence, or distant metastasis) or death from various causes. Survival results were calculated using Kaplan-Meier method and differences were compared using logarithmic rank test. We used receiver operating characteristic (ROC) curves to compare the sensitivity and specificity of UICC/AJCC rT staging and the rT staging recommended in this study for survival prediction. SPSS version 26.0 and R version 3.4.3 were used for all statistical analyses. Statistical significance was set at *p* < 0.05.

## Result

### Clinicopathological features

In a retrospective study of 175 rNPC patients treated with IMRT (Table 1), 139 were men and 36 were women. Age ≥ 45 years accounted for 66.29% (116/176) of patients. According to the pathological classification of NPC by the World Health Organization (WHO), there were 168 cases of WHO type III and 7 cases of WHO type I and II. According to the 8th UICC/AJCC based rT staging, the number of patients with rT1, rT2, rT3, and rT4 staging was 30, 67, 35, and 43, respectively. Among them, the proportion of smokers (60.0%) was more than that of non-smokers (40.0%), and the proportion of alcohol drinkers (24.0%) was less than that of non-drinkers (76.0%). 20.57% had a family history of cancer.

**Table 1:** Characteristics of 175 patients with rNPC

Parameter	*N*(%)
*Sex*	
Female	36(20.57)
Male	139(79.43)
*Age*	
≥ 45	116(66.29)
< 45	59(33.71)
*Pathological type*	
WHO type I, II	7(4.0)
WHO type III	168(96.0)
*Smoke*	
Yes	105(60.0)
No	70(40.0)
*Drink*	
Yes	42(24.0)
No	133(76.0)
*Time interval of initial recurrence*	
> 10	6(3.43)
≤ 10	169(96.57)
*Family history of cancer*	
Yes	36(20.57)
No	139(79.43)
*UICC/AJCC rT staging*	
rT1	30(17.14)
rT2	67(38.29)
rT3	35(20.00)
rT4	43(24.57)
*Recomandation rT staging*	
rT1	53(30.29)
rT2	44(25.14)
rT3	35(20.00)
rT4	43(24.57)

### UICC/AJCC rT stage survival

**Fig. 1:**
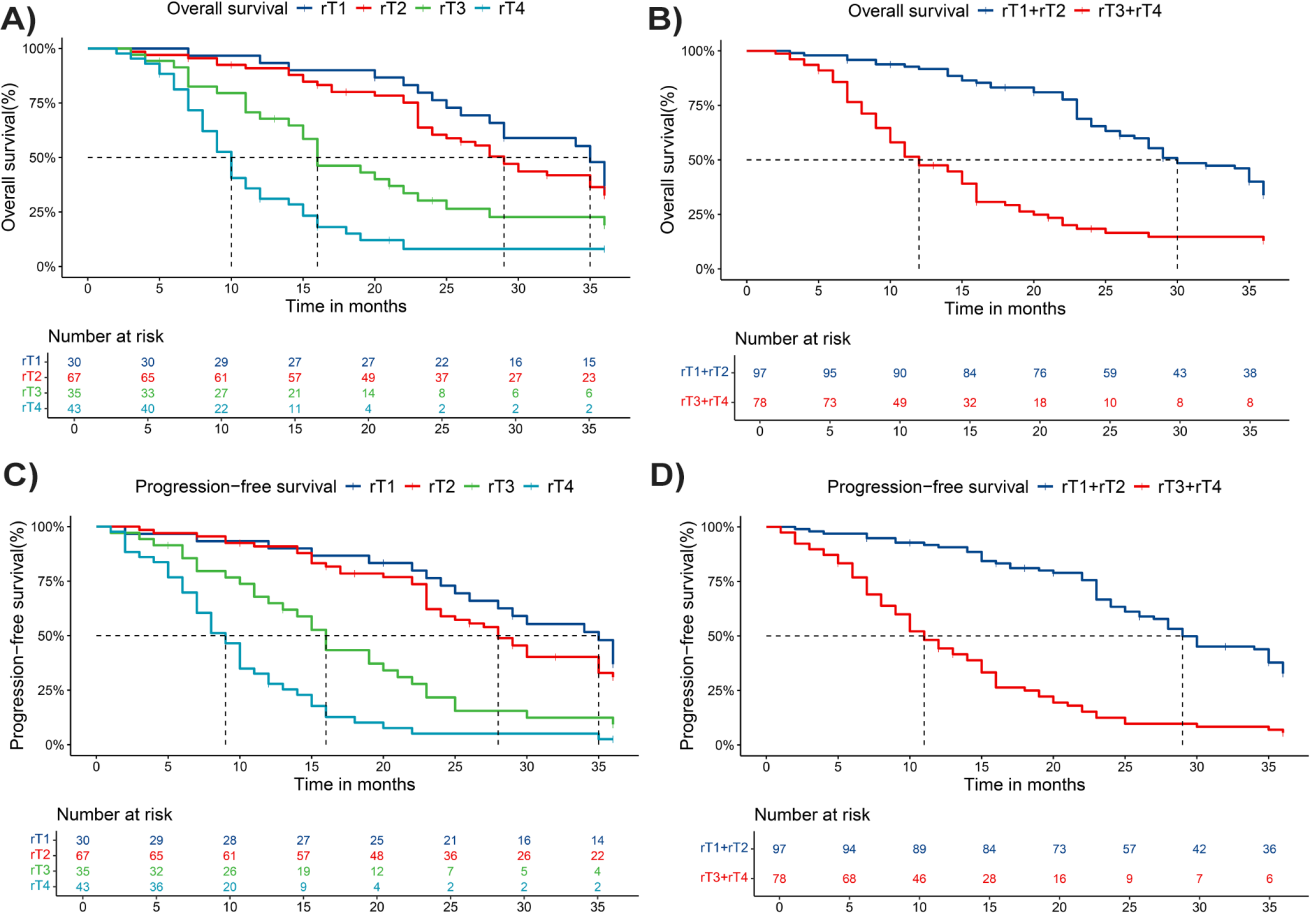
The 3-year survival rate in patients with rNPC according to the UICC/AJCC rT staging (**a**) OS of different rT classification (**b**) OS of advanced tumors versus early tumors (**c**) PFS of different rT classification (**d**) PFS of advanced tumors versus early tumors

According to the 8th UICC/AJCC rT staging, the 3-year OS of rT1, rT2, rT3 and rT4 were 29.98%, 27.09%, 19.44% and 12.32% respectively (Fig. 1a). Overall, the 3-year OS of patients with advanced tumors (rT3 and rT4) was 15.56%. It was significantly lower than 28.00% in early tumors (rT1 and rT2, *p* < 0.001) (Fig. 1b). Although patients with rT4 disease had significantly worse OS than patients with rT3 disease (*p* = 0.0020) and patients with rT3 disease had significantly worse OS than patients with rT2 disease (*p* = 0.0471), there was no significant difference between patients with rT1 and rT2 (*p* = 0.8059).

RT1, rT2, rT3 and rT4 period patients of 3-year PFS were 28.48%, 26.12%, 18.08%, 10.51%(Fig. 1c), patients with adanced cancer (rT3 and rT4) 3 years PFS was 13.89%, significantly lower than the early tumor (rT1 and rT2, *p* < 0.001) of 26.877%(Fig. 1d). 3-year PFS was significantly higher in patients with rT2 than in patients with rT3 (*p* = 0.002), and 3-year PFS was significantly higher in patients with rT3 than in patients with rT4 (*p* < 0.001). However, PFS at rT1 could not be distinguished from those at rT2 (*p* = 0.4045).

Therefore, we conclude that UICC/AJCC T staging is not suitable for staging management of treatment for rNPC and has significant limitations in predicting survival and grading risk management.

### Determine the effectiveness of rT staging recommended in this study

In the rT staging system of this study, based on previous literature research and clinical observations, it is recommended to divide patients involved only in the MP into stage T1. Therefore, 53, 44, 35 and 43 patients were involved in stage T1, T2, T3 and T4, respectively.

**Fig. 2:**
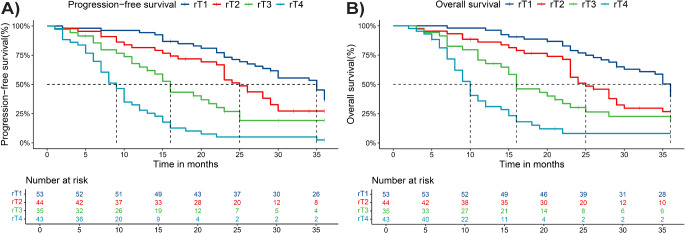
The 3-year survival rate in patients with rNPC according to new recommendation rT staging (**a**) OS of different rT classification (**b**) PFS of different rT classification

The 3-year OS of patients with rT1, rT2, rT3 and rT4 tumors was 30.44%, 24.91%, 19.44% and 12.32%. In addition, there were significant differences in survival between patients with rT1 and rT2 (*p* = 0.0229), rT2 and rT3 (*p* = 0.0458), and rT3 and rT4 (*p* = 0.0026)(Fig. 2a). 3-year PFS in rT1, rT2, rT3, and rT4 rNPC patients were29.12%, 24.03%,18.08% and 10.51%, respectively. Most importantly, we compared the PFS for rT1 and rT2, rT2 and rT3, and rT3 and rT4, respectively, and found that OS could be distinguished significantly (*p* = 0.0459, *p* = 0.0306, *p* = 0.0.0004, Fig. 2b).

**Fig. 3:**
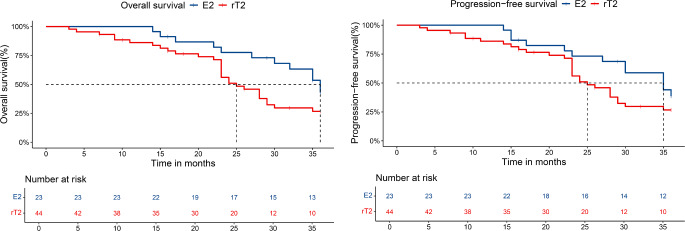
Comparisons of OS and PFS involved only the internal pterygoid muscle (E2) and remaining T2 patients(rT2)

Moreover, a comparison between the patients involved only the internal pterygoid muscle(E2) and the remaining T2 patients(rT2) showed that there was a significant difference in 3-year OS (*p* = 0.0498, Fig. 3a) and no significant difference in 3-yearPFS (*p* = 0.081, Fig. 3b). The 3-year OS of patients with E2 is significantly higher than that of patients with rT2, which may indicate that patients with E2 and rT2 should be divided into different periods.

Therefore, this study suggests that rT staging can effectively distinguish OS and PFS in rNPC patients according to different rT types, which has clinical significance.

### Comparison rT staging between UICC/AJCC and recommended in this study

**Fig. 4:**
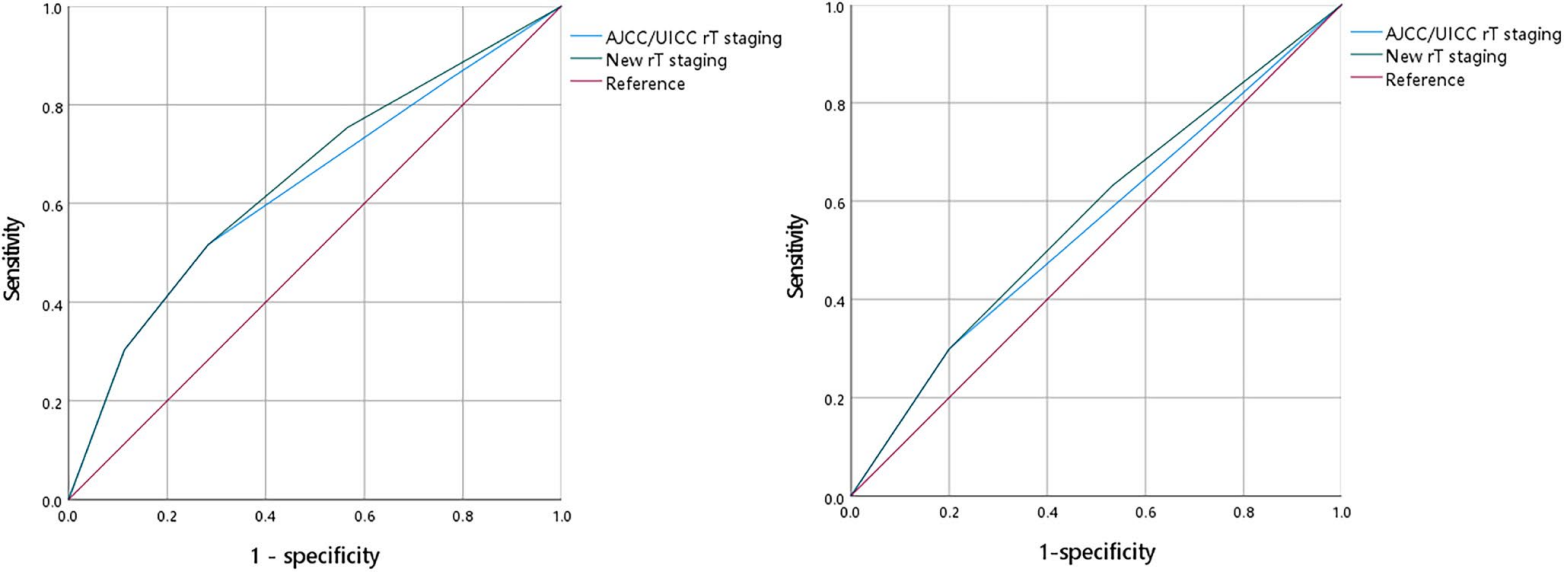
Comparisons of the sensitivity and specificity for the prediction of the survival using the UICC/AJCC rT staging and new recommendation rT staging(**a**) OS and (**b**) PFS

In ROC analysis and OS prediction, the rT staging system in this study showed higher prognostic value compared to the 8th UICC/AJCC rT staging system, with AUC values of 0.647 and 0.632, respectively (Fig. 4a). Similarly, rT staging in this study was more effective than UICC/AJCC rT staging in assessing PFS in rNPC patients (AUC: 0.566 vs. 0.548, Fig. 4b).

### Univariate and multivariate analyses of the effects of 3-year OS and 3-year PFS

Univariate analyses were performed to determine the effects of sex, age, alcohol consumption, smoking, family history, time interval of initial recurrence, and pathological tumor type on OS and PFS. The results showed that smoking, family history and time interval of initial recurrence had significant effects on 3-year OS and 3-year PFS (*p* < 0.05), while sex, age, alcohol consumption and pathological tumor type had no significant effects on 3-year OS and 3-year PFS (*p* > 0.05, Table 2).

We used the Cox proportional risk model to analyze the factors found to have a significant effect in univariate analysis (*p* < 0.05). Patients’ smoking, family history, and time interval of initial recurrence were used as regression covariates, and 3-year OS and 3-year PFS were specified as observational measures. Multivariate analysis showed that smoking, family history and first recurrence interval were independent prognostic factors for 3-year OS (*p* < 0.05). Smoking, family history, and first recurrence interval were independent prognostic factors for 3-year PFS (*p* < 0.05, Table 3).

**Table 2:** Univariate analysis of 175 cases of rNPC on OS and PFS

	3-year OS		3-year PFS	
	HR	*P* value	HR	*P* value
Sex	0.761	0.195	0.755	0.164
Age	1.201	0.361	1.212	0.327
Drink	1.255	0.272	1.276	0.218
Smoke	1.534	0.023^*^	1.746	0.003^*^
Family history of cancer	1.774	0.009^*^	1.941	0.001^*^
Time interval of initial recurrence	7.799	0.041^*^	8.753	0.031^*^
Pathological type	0.808	0.584	0.757	0.474

*Notes* * statistically significant with *p* < 0.05

**Table 3:** Multivariate analysis of 175 cases of rNPC on OS and PFS

	3-year OS		3-year PFS	
	HR	*P* value	HR	*P* value
Smoke	1.476	0.039^*^	1.682	0.005^*^
Family history of cancer	1.761	0.01^*^	1.925	0.002^*^
Time interval of initial recurrence	8.213	0.036^*^	9.414	0.026^*^

*Notes* * statistically significant with *p* < 0.05

## Conclusion

Compared with the 8th UICC/AJCC rT staging, the rT staging recommended in this study has a better predictive value for survival after treatment in patients with rNPC. This staging system can help clinicians provide patients with better precision radiation therapy and graded risk management. However, further research is needed to validate this new rT staging system.

## Discussion

For the 8th UICC/AJCC rT classification, many studies have found that survival rates for advanced tumors (rT3 and rT4) are significantly lower than for early tumors (rT1 and rT2), and that there is a statistically significant difference between the two classes. Our previous studies found significant differences in survival rates between rT4 and rT3, and between rT3 and rT2, while there was no significant difference in survival between patients with rT1 and rT2. In this series, we ruled out confounding factors for lymph node metastasis. And 3-year OS and 3-year PFS at rT1 were 29.98% and 28.48%, respectively, indistinguishable from 3-year OS and 3-year PFS at rT2 (27.09% and 26.12%, respectively). Therefore, 8th UICC/AJCC T staging may not be suitable for accurately predicting survival in patients with rNPC. In our study, we established a new rT staging version for rNPC therapy based on whether the tumor invaded the MP.

In our study, MRI detected that 59 out of 175 patients with rNPC had masticatory muscles(MM) involvement, and among these cases, only 23 patients were involved in MP. According to the 8th staging criteria, there was no significant significance in OS and PFS of patients in stage 1 and Stage 2. When patients with accumulated MP were included in stage 1 patients, and OS and PFS were recalculated, It showed significant differences in OS and PFS between newly classified stage 1 and stage 2 patients, and the ROC curve also showed that the new rT staging significantly improved the ability to predict OS and PFS.

At present, in NPC patients, there is a lot of debate about the degree of influence of masticatory muscle involvement such as MM, MP and the lateral pterygoid muscle (LP) LP on OS and PFS. On the one hand, the involvement of anatomical masticatory space affects the overall survival and local relapse-free survival of NPC patients. When the masticatory space involves both internal and external pterygoid muscles (Tang et al. 2010, Tang et al. 2014, Ng et al. 2008), such cases should be classified as stage T4. On the contrary, Luo et al. claimed that there was no significant difference in NPC patients with MP and LP involvement for nasopharyngeal carcinoma tumors involving mastigator space, while infratempral fossa involvement should be clearly distinguished from the above group (Luo et al. 2014). They suggested that in the future revision of TNM staging, MLPI should be graded T3, while IFI should be graded T4. Sze et al. also found that patients with MP and/or LP involvement had a similar prognosis to those with T1-T2 tumors (Sze et al. 2014). Therefore, they strongly recommend that NPC where the tumor only involves MP and/or LP should be classified as low-grade tumor stage. Pan et al. analyzed 1609 patients treated with IMRT from two clinical centers, analyzed and discussed the survival of 590 patients with MP and LP involvement, and found that 53 patients involved only MP and LP involvement, and these 53 patients did not involve other T3 and T4 standards (Pan et al. 2016). Their survival rate is significantly higher than that of patients with advanced disease. Therefore, they proposed a new T-staging system that relegates MP and LP to T2 (in line with the 8th edition of the UICC/AJCC NPC Staging System). These results minimize the confounding effects of T3 and T4 disease, but a major shortcoming of this study is that it did not separate the outcomes of patients with MP and LP. In contrast, in our current study, we found significant differences between patients with different levels of MM involvement.

Patients whose tumors only infiltrated MP or had T2 or T3 classifications had higher survival rates than those whose tumors invaded LP or T4 classifications. These results are consistent with those of Kang et al. and Zhang et al. Xiao et al (Kang et al. 2017, Zhang et al. 2014, Xiao et al. 2015). The two studies concluded that tumors infiltrating LP should be treated differently from tumors invading MP alone. Therefore, it seems reasonable that tumors that invade MP of rNPC should be classified as T1.

It’s worth noting, however, that the study has several limitations. First of all, this study suggests that rT staging is only applicable to patients with rNPC after radiotherapy. The main goal is to guide the survival and prognosis of patients with rNPC, and it can’t be used to guide the survival rate of patients with primary NPC after radiotherapy, chemotherapy or immunotherapy. Second, we included only patients who received radiation therapy for rNPC between 2012 and 2020, excluding those who underwent surgery. In fact, this study suggests that rT staging may also have a positive significance for staging of patients with rNPC undergoing surgical treatment, and predict their survival. This is a limitation of the study. In addition, this study is a single-center retrospective study, and clinical data from other hospital centers are needed to further external evaluation of the clinical validity of rT staging in this study.

## Data Availability

No datasets were generated or analysed during the current study.
